# Yangjing Capsule Extract Promotes Proliferation of GC-1 Spg Cells

**DOI:** 10.1155/2014/640857

**Published:** 2014-04-10

**Authors:** Zhiqiang Wang, Baofang Jin, Xindong Zhang, Yugui Cui, Dalin Sun, Chao Gao

**Affiliations:** ^1^Institute of Andrology, Nanjing University of Chinese Medicine, No. 138 Xianlin Road, Nanjing, Jiangsu 210023, China; ^2^State Key Laboratory of Reproductive Medicine, Clinical Center of Reproductive Medicine, First Affiliated Hospital, Nanjing Medical University, Nanjing 210029, China

## Abstract

*Objective*. To investigate the effect of Yangjing Capsule (YC) extract on proliferation of GC-1 spermatogonia (spg) cells and the mechanism. *Methods*. GC-1 spg cells were treated with 0.01, 0.1, and 1 mg/mL YC extract. MTT assay was performed to detect the cell viability. Flow cytometry was used to measure the cell cycle and apoptosis of GC-1 spg cells. Real-time PCR and western blot were applied to determine the mRNA and protein expression of Oct-4 and Plzf. Gfr**α**1 knockdown and LY294002 (PI3K inhibitor) were applied to explore the underlying mechanism. *Results*. After 48 h treatment of YC, the viability of GC-1 spg cells increased significantly and the ratio of apoptotic cells reduced significantly. The increased mRNA and protein expression of Oct-4 and Plzf suggested YC promoted self-renewal of GC-1 spg cells. Both Gfr**α**1 siRNAs and LY294002 treatments held back YC extract's stimulation effects on mRNA and protein expression of Oct-4 and Plzf and consequently inhibited the proliferation of GC-1 spg cells induced by YC extract. *Conclusion*. YC extract could stimulate the proliferation of GC-1 spg cells. Partly via Gfr**α**1, YC extract is able to trigger the activation of PI3K pathway and finally lead to self-renewal of GC-1 spg cells.

## 1. Introduction

About 15% of couples have problems in conceiving [1] and infertility beset around 80 million people worldwide by causing considerable psychological and financial burden. It is estimated that male reproductive dysfunction contributes to about half of infertile couples [2, 3]. Male infertility could be caused by various reasons, for instance, failure in spermatogenesis, epididymal maturation or storage of sperm, abnormal sperm delivery or accessory gland function, genetic or environmental factors, and sexual disorders [4, 5]. Among these causes, defects in spermatogenesis are the most common and account for 20%–25% of cases [6].

Spermatogenesis occurs in the seminiferous tubules of the testis with the seminiferous epithelium containing the developing germ cells and somatic Sertoli cells. Spermatogenesis is a continuous process by which the haploid germ cells are produced from differentiation of spermatogonial stem cells (SSCs) through mitosis and meiosis [7]. SSCs are responsible for sustaining fertility by tightly balancing between self-renewal and differentiation. With the establishment of balance, sperms are produced continually throughout the lifespan of an adult male [8–10]. Increased differentiation of SSCs at cost of self-renewal which destroys the balance would lead to male sterility [6]. Oct-4 and Plzf are considered to be responsible for preserving the pluripotency and self-renewal of stem cells [11, 12]. They are generally accepted as stem cell markers as they are downregulated during differentiation.

Self-renewal and differentiation of SSCs are strictly regulated by a combination of extrinsic gene signals from the niche as well as intrinsic signal pathways. Glial cell line-derived neurotrophic factor (Gdnf), secreted by Sertoli cells, is specifically responsible for the maintenance and self-renewal of SSCs [13, 14]. By binding to glycosylphosphatidylinositol- (GPI-) anchored cell surface molecule (Gfr*α*1), Gdnf is able to trigger PI3K/AKT pathway and eventually lead to self-renewal of SSCs [15, 16]. It has already been demonstrated that abnormal expression of extrinsic or intrinsic signaling molecules can give rise to impaired spermatogenesis [17, 18]. Oligozoospermia, asthenozoospermia, teratozoospermia, and azoospermia are the most common clinical manifestation of male infertility resulting from impaired spermatogenesis. In these cases, signal pathways regulating SSCs, such as PI3K/AKT pathway [19], are likely to be disrupted, thereby self-renewal of SSCs decreased and male infertility occurred.

The Yangjing Capsule (YC), which is composed of* Herba Epimedii Brevicornus, Placenta Hominis, Rhizoma Polygonati Sibirici, Radix Rehmanniae Preparata, Angelica sinensis,* and other components, has been suggested for the treatment of diseases of the reproductive system [20, 21]. Our previous clinical study found that YC was very effective for oligospermatism and can markedly increase sperm density for patients with a sperm density of over 5 × 10^6^/mL [22]. While medical practice has proved the efficacy of Chinese herbal formulation, the underlying molecular mechanisms of YC improving male fertility remain elusive. Our present study is aimed to determine whether YC could promote self-renewal of SSCs and explore the targeted signal pathways involved.

## 2. Materials and Methods

### 2.1. Chemicals

Dulbecco's modified eagle's medium (DMEM), fetal bovine serum (FBS), and lyophilized trypsin-EDTA were obtained from GIBCO BRL (Grand Island, NY, USA). 3-[4,5-Dimethylthiazolyl-2]-2,5-diphenyltetrazolium bromide (MTT), dimethyl sulfoxide (DMSO), sodium dodecyl sulfate (SDS), and Tris/HCl were purchased from Sigma (St. Louis, MO, USA). The whole protein extraction kits were purchased from Keygen (Keygen Biotech. Co. Ltd., Nanjing, China). Trizol reagent, PrimeScript RT Master Mix, and SYBR Green PCR Master Mix reagent kits were obtained from TaKaRa (TaKaRa Biotechnology, Dalian, China). The primers were synthesized by Invitrogen Life Tech (Carlsbad, CA, USA). Rabbit monoclonal anti-AKT (phospho S473), rabbit polyclonal anti-Plzf ab38739, and anti-Oct-4 ab18976 were from Abcam (Cambridge, MA, USA). Mouse monoclonal anti-*β*-actin was from Chemicon (Temecula, CA). Enhanced chemiluminescence was obtained from Amersham Biosciences (Uppsala, Sweden).

### 2.2. Preparation of YC Extract

The YC is composed of 11 traditional Chinese drugs: 13.3% Yinyanghuo (*Herba Epimedii Brevicornus*), 13.3% Muli (*Concha Ostreae (calcined)*), 13.3% Wangbuliuxing (*Semen Vaccariae Segetalis*), 10% Huangqi (*Radix Astragali Mongolici*), 10% Danggui (*Radix Angelicae Sinensis*), 6.7% Huangjing (*Rhizoma Polygonati Sibirici*), 6.7% Shayuanzi (*Semen Astragali Complanati*), 6.7% Ziheche (*Placenta Hominis*), 6.7% Shudihuang (*Radix Rehmanniae Preparata*), 6.7% Lizhihe (*Semen Litchi*), and 6.7% Shuizhi (*Hirudo*). The YC extract was prepared based on the methods described by Kao et al. [23]. The content of the YC (equivalent to 10 g of crude drug) was extracted with distilled water and subsequently subjected to ultrasonic extraction for 45 min. The supernatant was collected and centrifuged at 13,000 g and 4°C for 30 min to collect the supernatant, which was concentrated to 100 mL with a rotary evaporator at 60°C. The final concentration of the YC extract corresponded to 100 mg/mL of the crude herbal dose. The pH of the extract was adjusted to 7.0, and the extract was sterilized by filtration and stored at −80°C until use.

### 2.3. siRNA Transfection and PI3K Inhibition of GC-1 Spg Cells

GC-1 spg cells were cultured in DMEM, supplemented with 10% FBS, 50 IU/mL penicillin, and 50 *μ*g/mL streptomycin, and then incubated in a 5% CO_2_ incubator (Thermo Fisher Scientific, Rochester, USA) at 37°C. For determining effects of YC extract on the self-renewal, GC-1 spg cells were treated with 0, 0.01, 0.1, and 1 mg/mL YC extract and 20 ng/mL Gdnf (used as positive control), respectively. After 48 h, cells were collected for assay of proliferation, cell cycle, and apoptosis. RNAs and protein were prepared to detect the expression of Plzf and Oct-4 by quantitative PCR and western blot. To explore the underlying mechanism, Gfr*α*1 knockdown and PI3K inhibition were performed. For Gfr*α*1 knockdown, 19-nucleotide siRNA sequences (sense: 5′-GCC CUC ACA GGC UUC UGU U-3′ and antisense: 3′-CGG GAG UGU CCG AAG ACA A-5′) targeting mouse Gfr*α*1 sequence (GCC CTC ACA GGC TTC TGT T) were designed using BLOCK-iT RNAi Designer (Invitrogen) and synthesized by Invitrogen [24]. The Stealth RNAi negative control obtained from Invitrogen was used as a control for monitoring nonsequence-specific effects. 50 pmol Stealth RNAi negative control and Gfr*α*1 siRNA were transfected into GC-1 spg cells using Lipofectamine 2000 (Invitrogen) according to the manufacturer's instruction. At 48 h after transfection, cells were treated with 1 mg/mL YC extract for 48 h and collected for proliferation assay, quantitative PCR, and western blot. For PI3K inhibition, GC-1 spg cells were exposed by 50 *μ*M LY294002 (targeting the ATP-binding site of the PI3K) for abrogating PI3K activation. 2 h later, 1 mg/mL YC extract was added and cells were collected for proliferation assay, quantitative PCR, and western blot.

### 2.4. MTT Assay of Cell Proliferation

Cells were seeded in 96-well plate for 48 h treatment of 0, 0.01, 0.1, and 1 mg/mL YC extract, 20 ng/mL Gdnf, 50 *μ*M LY294002, and 50 pmol Gfr*α*1 siRNA, respectively (*n* = 5). MTT (5 mg/mL) was added with 20 *μ*L to each well and incubated for another 4 h before it was discarded. Then the purple-blue MTT formazan precipitate was dissolved in 100 *μ*L dimethyl sulfoxide (DMSO). The absorbance (OD) was measured at 490 nm. The proliferation ratio was calculated by the following formula: proliferation ratio (%) = (average  OD_treatment group_/average  OD_control group_ − 1) × 100%.

### 2.5. Cell Cycle and Apoptosis Assay

Cells were incubated in 6-well plates for 48 h treatment. Then cells were digested and washed. Cell cycle and apoptosis analysis were measured with flow cytometer (BD, Franklin, NJ, USA) according to the instructions of the Cycletest Plus DNA assay kit and Annexin V-FITC/PI apoptosis detection kit, respectively. The percentages of cells in G0/G1 phase, S phase, and G2/M phase were evaluated by the ModFit software (BD, Franklin, NJ, USA). The percentages of early stage and late stage apoptosis were evaluated with CellQuest software (BD, Franklin, NJ, USA). Total apoptosis rate was equal to early stage apoptosis plus late stage apoptosis.

### 2.6. RNA Isolation and Real-Time PCR

Cells at a density of 4 × 10^5^/well were plated in 6-well plates for 48 h treatment. The total RNA was extracted using Trizol reagent, measured by spectrometry at an OD260/280, and reverse transcribed into cDNA in a total volume of 20 *μ*L with PrimeScript RT Master Mix. All of the RT-PCR reactions were performed with a CFX96 real-time PCR system (Bio-Rad Laboratories, Hercules, CA) using SYBR Green chemistry (Bio-Rad Laboratories). GAPDH was selected as the control. The primer sequences were as follows: GAPDH, sense: 5′-AGG TTG TCT CCT GCG ACT TCA-3′ and antisense: 5′-GGG TGG TCC AGG GTT TCT TAC T-3′; Plzf, sense: 5′-CAC ACA GGC AGA CCC ATA CT-3′ and antisense: 5′-TTT GTG GCT CTT GAG TGT GC-3′; OCT-4, sense: 5′-CTT GCT GCA GAA GTG GGT GGA GGA A-3′ and antisense: 5′-CTG CAG TGT GGG TTT CGG GCA-3′ [25, 26]. The reactions were performed at 94°C for 3 min followed by 40 cycles at 94°C for 30 s, 55°C for 30 s, and 72°C for 30 s. The final extension was carried out for 5 min at 72°C. A melting curve analysis was performed to confirm the products. The relative abundances of the target mRNAs were calculated using the 2^−ΔΔCt^ method. The data were expressed as the percentage of control (100%).

### 2.7. Protein Extraction and Western Blot Analysis

Cells were seeded in 60 mm dishes at a density of 1 × 10^6^/well for 48 h treatment. The cells were harvested, washed three times with precooled PBS, and treated with cell lysis buffer for western blot analysis. After centrifugation at 12.000 g at 4°C for 20 min, the supernatants were collected and stored at −80°C until analysis.

The concentrations of protein were measured by the Bio-Rad Bradford assay (Bio-Rad Laboratories, Hercules, CA). The proteins were normalized to 50 *μ*g/lane, separated by 12% sodium dodecyl sulfate polyacrylamide gel electrophoresis (SDS-PAGE), and subsequently transferred to nitrocellulose membranes. After treatment with blocking solution (5% skim milk powder in Tris-buffered saline) at 37°C for 1 h, the membranes were incubated overnight with the primary antibodies rabbit monoclonal anti-pAKT (1 : 5000), rabbit polyclonal anti-Plzf (1 : 500), rabbit polyclonal anti-Oct-4 (1 : 400), or mouse monoclonal anti-*β*-actin (1 : 5000) at 4°C. After washing with TBS three times, the membranes were incubated with HRP-conjugated secondary antibodies (1: 5000) at 37°C for 1 h and examined using enhanced chemiluminescence. The relative protein levels in each sample were normalized to the levels of *β*-actin to standardize the variations in loading. Densitometric analyses of the scanned immunoblotting images were performed using a Quantity One image system. The data are expressed as a percentage of the control (100%).

### 2.8. Statistical Analysis

Data were analyzed using a SPSS 16.0 statistical package. All results were expressed as mean ± standard deviation (S.D.). One-way analysis of variance (ANOVA) was used to analyze the difference between groups, followed by Dunnett's *t*-test. *P* < 0.05 was considered as statistically significant.

## 3. Results

### 3.1. Effects of YC Extract on Proliferation of GC-1 Spg Cells

**Figure 1:**
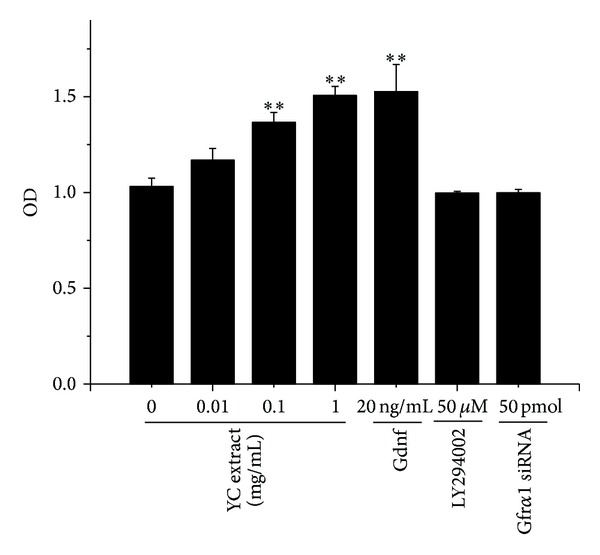
Effects of YC extract on proliferation of GC-1 spg cells. Cells were treated with 0, 0.01, 0.1, and 1 mg/mL YC extract, 20 ng/mL Gdnf, 50 *μ*M LY294002, and 50 pmol Gfr*α*1 siRNA, respectively. **Significantly different from control at *P* < 0.01, *n* = 5.

The effects of YC extract on cell proliferation are shown in Figure 1. Gdnf, responsible for proliferation of SSCs, was used as positive control. In the presence of 0.01, 0.1, and 1 mg/mL YC extract for 48 h, OD values increased in a dose dependent manner. The proliferation rates of GC-1 spg cells increased by 13.38%, 32.47%, and 46.04%, respectively. In the presence of 20 ng/mL Gdnf for 48 h, the proliferation rates of GC-1 spg cells significantly increased by 47.98%. There are no evident changes observed with the treatment of 50 *μ*M LY294002 or 50 pmol Gfr*α*1 siRNA.

### 3.2. Effects of YC Extract on Cell Cycle of GC-1 Spg Cells

**Figure 2:**
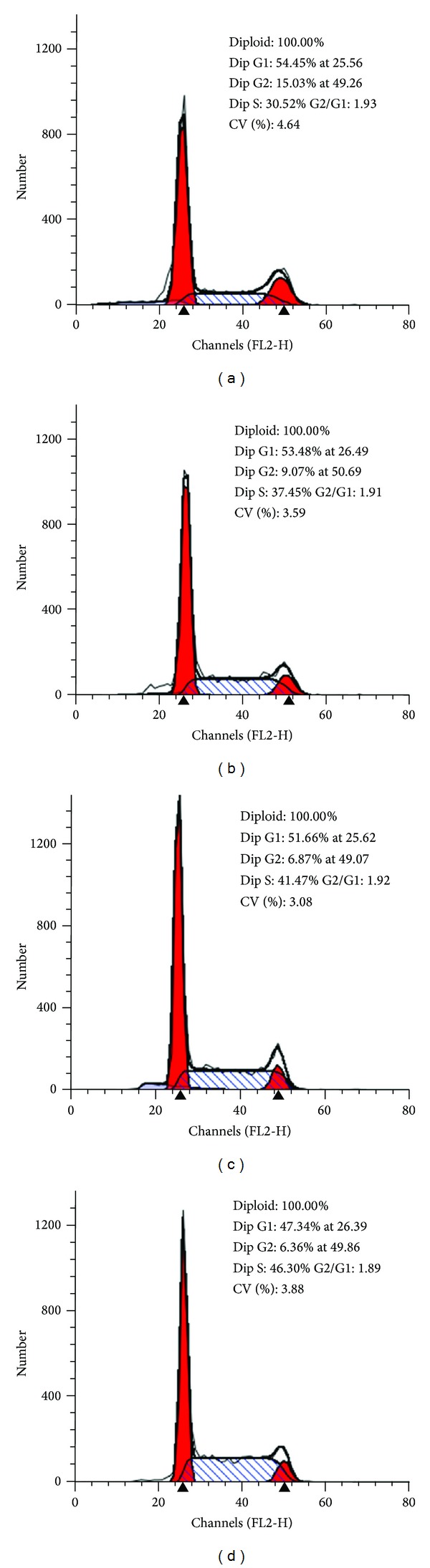
Effects of YC extract on cell cycle of GC-1 spg cells. Cells were treated with 0 (a), 0.01 (b), 0.1 (c), and 1 (d) mg/mL YC extract for 48 h, respectively. The analysis was performed in triplicate and representative data was shown.

Results of cell cycle assay of GC-1 spg cells are shown in Figure 2. The percentage of S phase in control group was 30.52% (Figure 2(a)), and after 48 h treatment of different doses of YC extract, the percentage of S phase evidently rose to 37.45%, 41.47%, and 46.30%, respectively (Figures 2(b), 2(c), and 2(d)). A decline of percentage of G0/G1 phase was also observed. These data suggested that YC extract could advance the cell cycle from the G1 phase to the S phase and promote proliferation of spg cells.

### 3.3. Effects of YC Extract on Apoptosis of GC-1 Spg Cells

**Figure 3:**
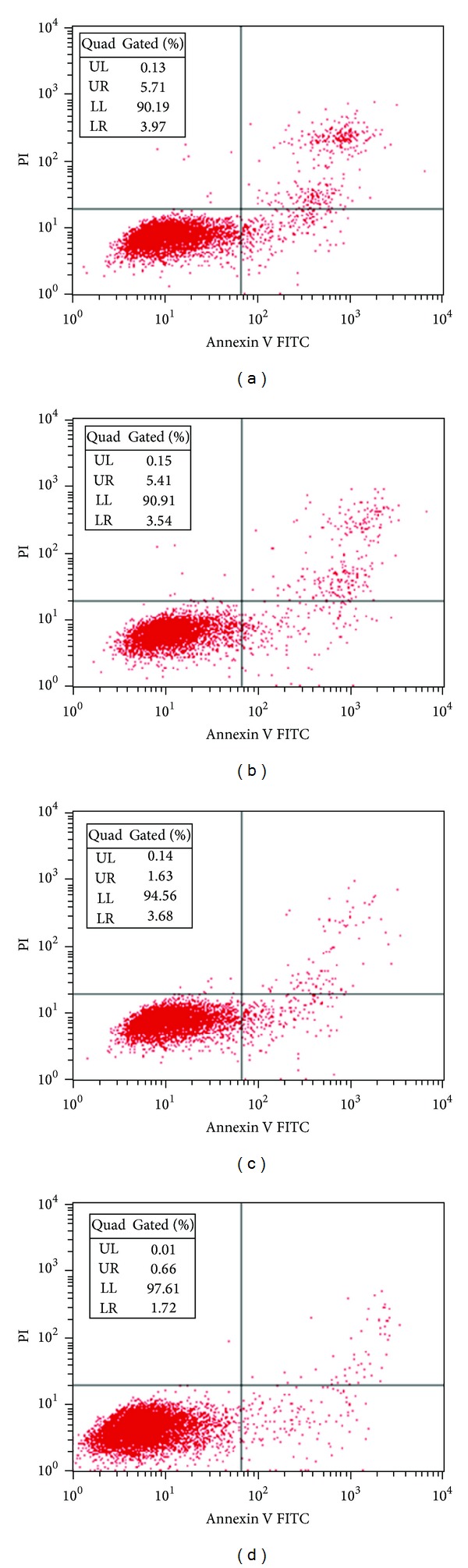
Effects of YC extract on apoptosis of GC-1 spg cells. Cells were treated with 0 (a), 0.01 (b), 0.1 (c), and 1 (d) mg/mL YC extract for 48 h, respectively. Annexin V^+^/PI^−^ population (LR in diagram) indicated early apoptosis, and annexin V^+^/PI^+^ population (UR in diagram) indicated late apoptosis. The analysis was performed in triplicate and representative data was shown.

As shown in Figure 3, total apoptosis rate was equal to early stage apoptosis (LR) plus late stage apoptosis (UR). The apoptosis rates are 9.68 (a), 8.95 (b), 5.31 (c), and 2.38 (d), respectively. 0.01 mg/mL YC extract had no evident effect on apoptosis of GC-1 spg cells. However, 0.1 and 1 mg/mL YC extract notably inhibited cell apoptosis.

### 3.4. Effects of the YC Extract on the Expression of Oct-4 and Plzf mRNAs and Proteins

**Figure 4:**
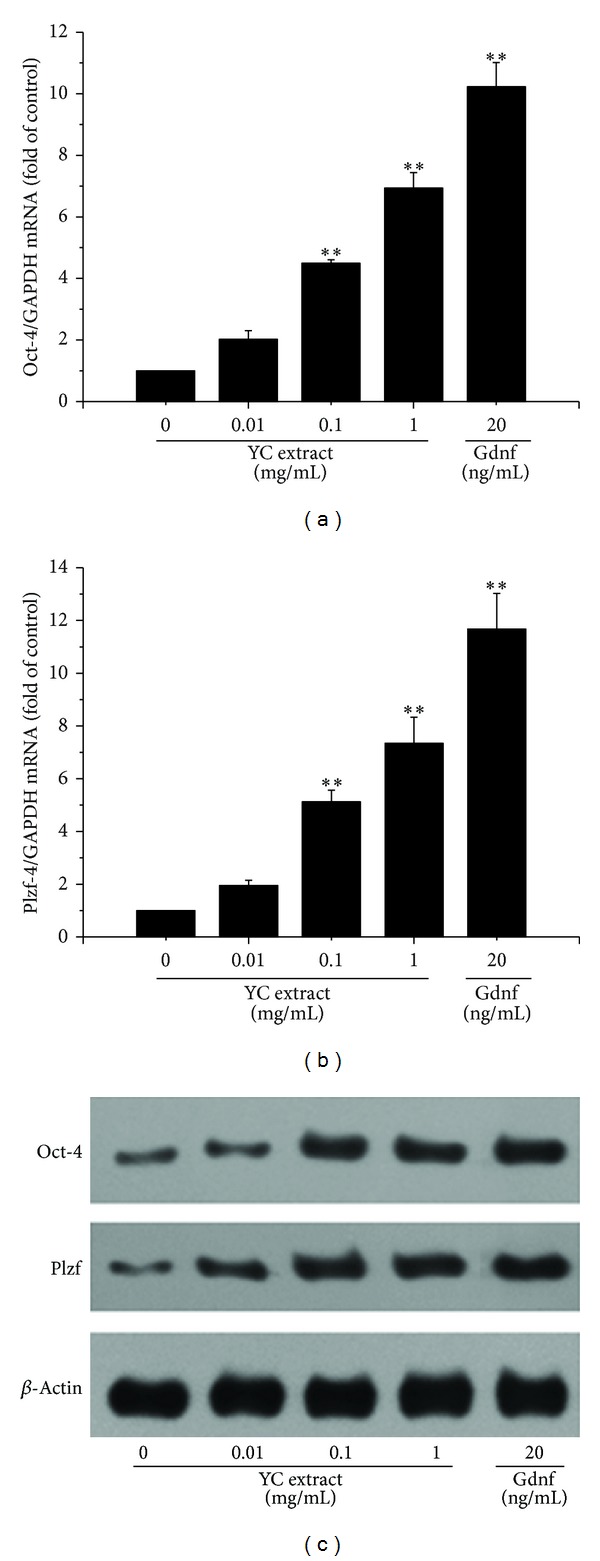
Effects of the YC extract on the expression of Oct-4 and Plzf mRNAs and proteins in GC-1 spg cells. GC-1 spg cells were treated with 0.01, 0.1, and 1 mg/mL YC extract or 20 ng/mL Gdnf for 48 h. Expression of mRNAs was detected by real-time PCR. Expression of proteins was detected by western blot. The data are expressed as the percentage of the control (100%). **Significantly different from control at *P* < 0.01. The analysis was performed in triplicate and representative bands were shown.

Oct-4 and Plzf were chosen as stem cell markers to confirm the undifferentiated status of GC-1 spg cells. Figures 4(a) and 4(b) showed that the expression of Oct-4 and Plzf mRNAs increased significantly by exposure of 0.1, 1 mg/mL YC extract and 20 ng/mL Gdnf. Accordingly, Figure 4(c) showed that the expression of Oct-4 and Plzf proteins increased evidently at the same condition. The result suggested that YC extract could promote self-renewal of GC-1 spg cells.

### 3.5. Gfr*α*1 Knockdown or PI3K Inhibition Blocks YC Extract Induced Proliferation of GC-1 Spg Cells

**Figure 5:**
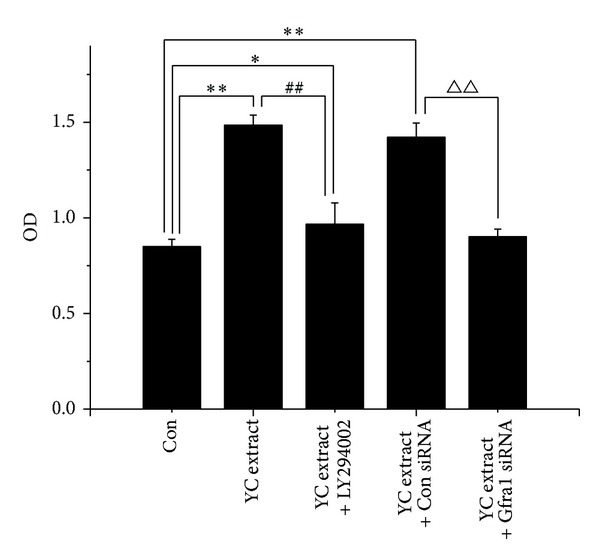
Gfr*α*1 knockdown or PI3K inhibition blocks YC extract induced proliferation of GC-1 spg cells. GC-1 cells were exposed to control blank, 1 mg/mL YC extract, 1 mg/mL YC extract with 50 *μ*M LY294002, and 1 mg/mL YC extract with or without 50 pmol Gfr*α*1 siRNA. *P < 0.05; **P < 0.01 compared with control group; ^##^ *P* < 0.01 compared with YC extract group; ^△△^ *P* < 0.01 compared with YC extract and control siRNA group, *n* = 5.

To investigate the mechanism of YC extract induced proliferation of GC-1 spg cells, Gfr*α*1 knockdown and PI3K inhibition were performed. As shown in Figure 5, YC extract significantly stimulated the cell proliferation. However, both Gfr*α*1 siRNA and LY294002, an inhibitor of PI3K, could evidently abrogate the stimulative effect of YC extract.

### 3.6. Gfr*α*1 Knockdown or PI3K Inhibition Blocks YC Extract Induced Upregulation of Oct-4 and Plzf Expression

**Figure 6:**
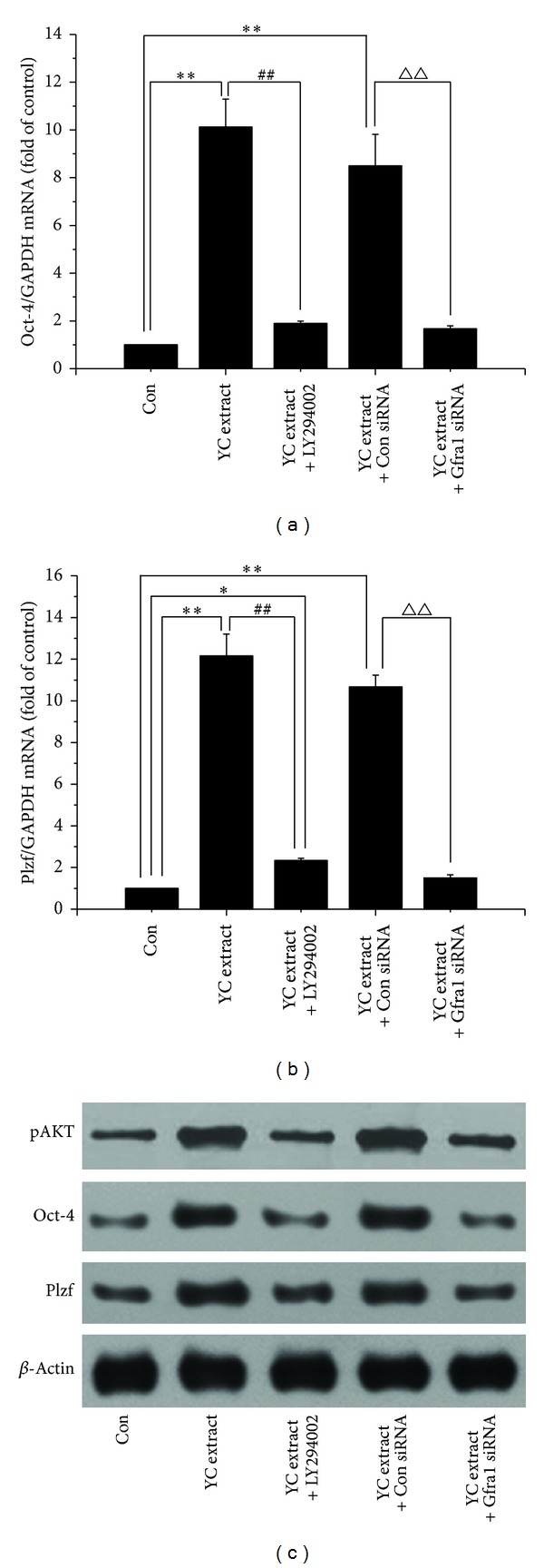
Gfr*α*1 knockdown or PI3 K inhibition blocks YC extract induced upregulation of Oct-4 and Plzf expression. GC-1 cells were exposed to control blank, 1 mg/mL YC extract, 1 mg/mL YC extract with 50 *μ*M LY294002, and 1 mg/mL YC extract with or without 50 pmol Gfr*α*1 siRNA. *P < 0.05; **P < 0.01 compared with control group; ^##^ *P* < 0.01 compared with YC extract group; ^△△^ *P* < 0.01 compared with YC extract and control siRNA group. The analysis was performed in triplicate and representative bands were shown.

The results of Figure 6 showed that both Gfr*α*1 siRNA and LY294002 could abolish the upregulation of Oct-4 and Plzf expression at levels of mRNA and protein induced by YC extract. Meanwhile, upregulation of pAKT protein expression induced by YC extract, evidence for PI3K pathway activation, was also suppressed by Gfr*α*1 siRNA and LY294002. Combined with the results above, it could be inferred that Gfr*α*1 and PI3K played crucial roles in the promotion of self-renewal induced by YC extract.

## 4. Discussion

YC extract has been used for male infertility therapy in clinic for years, but the mechanisms still remain unclear. Our results suggested that YC extract could stimulate self-renewal of SSCs and protect SSCs from apoptosis, thus improving spermatogenesis.

Oct-4 is considered to be the master transcription factor responsible for preserving the pluripotency and self-renewal of stem cells [11, 25]. It is downregulated during differentiation with loss of pluripotency [27–29] and generally accepted as a stem cell marker. Plzf has also drawn wide attention due to its role of regulating differentiation. Plzf is claimed to be essential for maintaining pluripotent properties and self-renewal as its expression is confined to stem cells and early progenitor cells [12, 30, 31]. While Plzf is expressed at high level in undifferentiated pluripotent stem cells, its expression declined when differentiation started. Our results showed that 48 h treatment of YC extract not only promoted the proliferation of GC-1 spg cells but also promoted self-renewal of cells proved by enhanced expression of Oct-4 and Plzf mRNAs and proteins, which were quite similar to the effect of Gdnf treatment (Figures 1 and 4). As a matter of course, we deduced that YC extract probably functioned in the similar ways as Gdnf.

Gdnf, secreted by Sertoli cells, is so far the only known paracrine factor specifically responsible for the maintenance and self-renewal of SSCs* in vivo* [13, 14]. Lin et al. reported that Gdnf is a member of the transforming growth factor beta (TGF-*β*) superfamily [32]. Gdnf signals through a multicomponent receptor complex are composed of the Ret receptor tyrosine kinase and Gfr*α*1 [33, 34]. Gdnf can trigger various downstream signal pathways to promote cell survival and self-renewal via GPI-linked protein Gfr*α*1 [35, 36]. Based on the comparable effects of YC extract and Gdnf, we speculated that Gfr*α*1 also plays a key role in YC extract's biological effects. To confirm our hypothesis, we used siRNA to knockdown Gfr*α*1 expression in current research. Figure 5 showed that Gfr*α*1 knockdown almost entirely abolished the proliferous effect of GC-1 cells induced by YC extract. Corresponding with this, Gfr*α*1 knockdown also abrogated the elevated expression of Oct-4 and Plzf mRNAs and proteins induced by YC extract (Figure 6). On the basis of these results, we inferred that YC extract exerts biological effects partly via Gfr*α*1.

To further investigate the mechanism of YC extract, we treated GC-1 cells with 50 *μ*M LY294002 to inhibit PI3K pathway before the treatment of YC extract. PI3K pathway has been demonstrated to play a central role in the Gdnf induced self-renewal of SSCs [37, 38]. The binding of Gdnf to Gfr*α*1 triggers phosphorylation of SRC-kinase family proteins followed by activation of PI3K/AKT pathway and eventually leads to self-renewal of SSCs via expression of* N-myc* gene [15, 16]. As has been stated, like Gdnf, YC extract also induces self-renewal via Gfr*α*1, and activation of PI3K pathway is most likely very crucial in YC extract's effects. As expected, pAKT protein expression increased evidently after treatment of YC extract standing for PI3K pathway activation (Figure 6). PI3K pathway inhibition evidently removed the proliferous effect of GC-1 spg cells induced by YC extract which was shown in Figure 5. Consistent with this, the upregulated Oct-4 and Plzf mRNAs and proteins also dropped down by LY294002 treatment (Figure 6).

In summary, YC extract could improve spermatogenesis by means of promoting self-renewal of SSCs and protecting SSCs from apoptosis, which were verified by cell proliferation and upregulation of OCT-4 and Plzf expression after the treatment of YC extract. To explore possible mechanism, we performed Gfr*α*1 knockdown and PI3K inhibition before application of YC extract. The results showed that both Gfr*α*1 knockdown and PI3K inhibition were able to evidently remove the cell proliferation and upregulation of OCT-4 and Plzf expression induced by YC extract. Therefore, we concluded that, partly via Gfr*α*1, YC extract could trigger the activation of PI3K pathway and finally lead to self-renewal of SSCs. However, as YC extract is composed of multiple components, further studies are needed to identify the primary effective component.
